# Semiconductor Hyperbolic Metamaterials at the Quantum Limit

**DOI:** 10.1038/s41598-018-35099-8

**Published:** 2018-11-12

**Authors:** Inès Montaño, Salvatore Campione, John F. Klem, Thomas E. Beechem, Omri Wolf, Michael B. Sinclair, Ting S. Luk

**Affiliations:** 1 Sandia National Laboratories, Albuquerque, USA; 2 Center for Integrated Nanotechnologies, Sandia National Laboratories, Albuquerque, USA; 3 Present Address: Department of Physics and Astronomy, Northern Arizona University, Flagstaff, USA

## Abstract

We study semiconductor hyperbolic metamaterials (SHMs) at the quantum limit experimentally using spectroscopic ellipsometry as well as theoretically using a new microscopic theory. The theory is a combination of microscopic density matrix approach for the material response and Green’s function approach for the propagating electric field. Our approach predicts absorptivity of the full multilayer system and for the first time allows the prediction of in-plane and out-of-plane dielectric functions for every individual layer constructing the SHM as well as effective dielectric functions that can be used to describe a homogenized SHM.

## Introduction

Hyperbolic metamaterials (HMs)^1–3^ are a special class of metamaterials made of metallodielectric multilayers that have been under intense investigation recently because of the extreme anisotropy that can be created artificially^4–9^. Possible implementations include alternating subwavelength layers of positive and negative permittivity materials^4^, nanowire arrays^10,11^, and multilayer graphene^12–14^. Optical phase diagrams show that HMs can behave as effective dielectric (${\varepsilon }_{\perp },{\varepsilon }_{\parallel } > 0$), effective metal (${\varepsilon }_{\perp },{\varepsilon }_{\parallel } < 0$), or exhibit Type I (${\varepsilon }_{\perp } < 0,{\varepsilon }_{\parallel } > 0$), or Type II (${\varepsilon }_{\parallel } < 0,{\varepsilon }_{\perp } > 0$) hyperbolic character, where ${\varepsilon }_{\parallel },$ and ${\varepsilon }_{\perp }$ denote in-plane and out-of-plane effective medium dielectric functions, respectively. The frequency dependent optical properties of an HM depend on the filling factor (i.e. the ratio of metal/conducting media to dielectric) as well as the optical properties of the metallic and dielectric layers. Hyperbolic metamaterials can have extremely large densities of states (infinite in the lossless effective medium limit) which can greatly enhance spontaneous emission^15–19^, enhance near-field thermal energy transfer^20,21^, and lead to enhanced absorption processes^22,23^. Because at mid-infrared frequencies highly doped semiconductors behave like metals (${\varepsilon }_{\perp },{\varepsilon }_{\parallel } < 0$), it has recently been discovered that semiconductor hyperbolic metamaterials (SHMs) can be fabricated using epitaxial growth of alternating layers of deeply sub-wavelength doped and undoped semiconductor layers^4,24,25^. SHMs offer unprecedented control of carrier concentration, layer thicknesses, and interface smoothness when compared to conventional metal/dielectric counterparts^26,27^, and also feature higher carrier mobilities. With epitaxial growth, highly doped layers can be as thin as few nanometers which enables this class of metamaterials to support very large wave momentum and hence large photonic density of states.

Of key importance for light-matter interaction physics and device applications of HMs is the actual determination of the effective permittivities of the hyperbolic metamaterial where the specific multilayer structure of the HMs is replaced with an *effective medium* with uniform properties in each direction. Effective medium theories have become ubiquitous and extraction or prediction of effective permittivities of multilayered materials has been a topic of intense research for quite some time^28–34^. While effective permittivities of traditional metallodielectric multilayers can generally be calculated in a straightforward way, e.g. see^30^, the situation is different for SHMs, particularly SHMs at the quantum limit. In SHMs the metal layers are replaced by highly doped semiconductor layers that although they behave like a metal cannot necessarily be described by an isotropic Drude model as it would be appropriate for a metal layer. In nanoscale semiconductor multilayer structures the composite physical properties are determined by the eigenstates (wavefunctions and eigen energies) of the system which in turn are determined by the nanoscale dimensions and relative placement of the layers and not the bulk properties. As a result, the electromagnetic response of a quantum well can be altered dramatically through bandstructure engineering and can depend strongly on confinement effects, doping, and many-body interactions. An example for the extreme tunability was given in^35^, where the crossover from confined plasmon mode (Berreman mode, isotropic Drude permittivity), to multisubband plasmon (anisotropic permittivity), to intersubband (ISB) plasmon (highly anisotropic permittivity) was achieved by decreasing the thickness of a highly doped quantum well. This tunability of the light-matter coupling through bandstructure engineering is the reason why electromagnetic modeling of SHMs is not as straightforward as that of metallodielectric HMs. In SHMs the highly doped quantum well layer can have a very anisotropic electromagnetic response which has to be quantized in order to describe the SHMs through effective permittivities.

In this article, we present a new theory that combines a microscopic density matrix approach for the material response and a Green’s function approach for the propagating electric field. This approach allows us for the first time to predict in-plane and out-of-plane dielectric functions for every individual layer constructing the SHM as well as effective dielectric functions for a homogenized SHM.

## Methods

### Effective conductivity model

Studies of highly doped quantum wells have shown that the Coulomb interaction can yield an effective coupling of all active intersubband excitations that causes the excitation of a unique collective mode, the multisubband plasmon. Previous attempts to quantize the electromagnetic response of highly doped quantum wells have focused on capturing this effect (called the depolarization effect) by using an effective out-of-plane conductivity, ${\tilde{\sigma }}_{\perp }$, which approximates the impact of the depolarization effect on the light-interaction^35–39^. In the following we will refer to these models as effective conductivity models (ECMs). In an effective conductivity model, the optical absorption of a single quantum well per unit area is approximated using

1 $${A}_{SQW}(\omega )=\frac{1}{2}{\rm{Re}}[{\int }_{-\infty }^{\infty }{J}_{z}(z,\omega ){E}_{z}^{\ast }(z,\omega )dz]$$

2 $$\approx \,\frac{1}{2}{\rm{Re}}[{\tilde{\sigma }}_{\perp }(\omega )|{E}_{0}(\omega {)|}^{2}]\,,$$

where $z$ is along the out-of-plane direction, ${J}_{z}(z,\omega )$ is the z-component of the current density, ${E}_{z}(z,\omega )$ the z-component of the total electric field, and ${E}_{0}(\omega )$ the external field. Note, that in Eq. 2 ${\tilde{\sigma }}_{\perp }$ describes only the nonretarded response of the electric current to the external field, ${E}_{0}(\omega )$, not the response to the total field, ${E}_{z}(z,\omega )$^38^. In this framework the single quantum well is represented by an anisotropic layer with an effective thickness ${L}_{eff}$ that is characterized by a spatially uniform out-of-plane permittivity. This spatially uniform out-of-plane permittivity is calculated by relating the out-of-plane conductivity of the doped quantum wells, ${\sigma }_{\perp }$, to the corresponding effective conductivity^35,36,39^

3 $${\varepsilon }_{\perp }^{ECM}(\omega )={\varepsilon }_{r}\frac{{\sigma }_{\perp }(\omega )}{{\tilde{\sigma }}_{\perp }(\omega )}={\varepsilon }_{r}(1-\sum _{n}\frac{{\omega }_{p,n}^{2}}{{\omega }^{2}-{\omega }_{n}^{2}+{\rm{i}}\omega \gamma })\mathrm{.}$$

In Eq. 3 ${\varepsilon }_{r}$ is the relative background permittivity, ${\omega }_{n}$ is the transition frequency and ${\omega }_{p,n}$ the plasma frequency associated with the intersubband transition $n\to n+1$ between consecutive subbands. The plasma frequency can be expressed as

4 $${\omega }_{p,n}^{2}=\frac{{f}_{n}{e}^{2}{\rm{\Delta }}{N}_{n}}{{m}^{\ast }{\varepsilon }_{0}{\varepsilon }_{r}{L}_{eff,n}}\,,$$

where ${f}_{n}$ is the oscillator strength, ${\rm{\Delta }}{N}_{n}={N}_{n+1}-{N}_{n}$ is the difference in sheet carrier density, and ${L}_{eff,n}$ the corresponding effective length^36^. The two main advantages of this approach are that it allows a) quantization of the electromagnetic response through a simple formula and b) easy generalization to account for nonparabolic subbands. However, it is important to note that ${\varepsilon }_{\perp }^{ECM}$ is not the effective permittivity of the metamaterial. Instead ${\varepsilon }_{\perp }^{ECM}$ only describes the approximate optical response of a homogeneous slab of quantum well material with an effective thickness ${L}_{eff}$ that can differ quite substantially from the physical quantum well thickness^38^. To complicate matters even further, Eq. 4 shows that the slab can not necessarily be described by one effective thickness, since each active transition can have its own effective length. This creates an ambiguity regarding how to practically calculate an effective permittivity of the metamaterial from ${\varepsilon }_{\perp }^{ECM}$.

We show in the following that for certain SHMs, the effective-conductivity model can be used to approximate the permittivity of the quantum well layer using ${\varepsilon }_{\perp }^{QW}\approx {\varepsilon }_{\perp }^{ECM}$, i.e. approximating ${L}_{eff,n}={L}_{QW}$. In this case ${\varepsilon }_{\perp }^{ECM}$ can then be used to calculate the electromagnetic response using the transfer-matrix approach or to calculate effective out-of-plane permittivities using the standard anisotropic effective medium approximation. However, this approach has to be used with caution as it will ultimately fail if the SHM contains more complicated quantum well structures as e.g. double quantum well structures or even single quantum wells where either the optical response is not dominated by next-neighbor transitions^40^ or the effective lengths differ too strongly from the physical quantum well thickness.

### Spectroscopic ellipsometry method

We have recently presented an experimental approach to extract the needed permittivities of SHMs using spectroscopic ellipsometry^34^. Fitting measured ellipsometry data we obtained the anisotropic permittivities of the individual quantum well and barrier layers and then computed effective in-plane and out-of-plane dielectric functions of SHMs by employing the anisotropic effective medium theory (for details see^34^). As we have shown in^34^, employing either the individual permittivities using a transfer-matrix approach (anisotropic superlattice model) or instead employing the effective permittivities using the anisotropic effective medium approximation (anisotropic effective medium model) both allowed us to successfully recover spectral as well as angular properties of measured absorptivity/emissivity spectra of the considered SHM^32^.

### Microscopic model

In this work, we now present a corresponding theoretical approach to determinate the electromagnetic response of SHMs. The presented theory is a combination of microscopic density matrix approach for the material response and Green’s function approach for the propagating electric field in the structure and allows us to predict the absorptivity of the SHM as well as permittivities of the individual quantum well and barrier layers (anisotropic superlattice model) and effective in-plane and out-of-plane dielectric functions for the homogenized SHM (anisotropic effective medium model) in excellent agreement with experimental data obtained from ellipsometry measurements. Additionally, our theory shows that measured absorptivity spectra alone are insufficient in confirming the accuracy of extracted or calculated effective permittivities as these measurements are not sensitive enough to unambiguously capture the detailed electromagnetic response of SHMs.

In our model the local electric field in the n-th layer of a SHM is determined by solving the inhomogeneous self-consistent integral equation

5 $$\begin{array}{rcl}{{\bf{E}}}^{(n)}(z) & = & {E}_{\mathrm{0,}+}{\hat{{\bf{e}}}}_{+}^{(n)}{{\rm{e}}}^{{\rm{i}}{q}_{\perp }^{(n)}z}+{E}_{\mathrm{0,}-}{\hat{{\bf{e}}}}_{-}^{(n)}{{\rm{e}}}^{-{\rm{i}}{q}_{\perp }^{(n)}z}\\  &  & -{\rm{i}}{\mu }_{0}\omega \sum _{m\mathrm{=1}}^{N}\,\int \,dz^{\prime} \,\int \,dz^{\prime\prime} \overleftrightarrow{\tilde{G}}(z,z^{\prime} )\cdot {\overleftrightarrow{\sigma }}^{(m)}(z^{\prime} ,z^{\prime\prime} )\cdot {{\bf{E}}}^{(m)}(z^{\prime\prime} ),\end{array}$$

where ${\hat{{\bf{e}}}}_{\pm }^{(n)}={\hat{{\bf{p}}}}_{\pm }^{(n)}({\hat{{\bf{s}}}}^{(n)})$ for p-polarized (s-polarized) light and ${E}_{\mathrm{0,}\pm }$ denote the corresponding amplitudes of the incident field ${{\bf{E}}}_{0}$ and its reflections at the various interfaces. $\overleftrightarrow{\tilde{G}}(z,z^{\prime} )$ is the retarded Green’s function tensor modified to describe a superlattice in the presence of a general multilayer geometry (air, active medium, substrate, etc.). The nonlocal and nonuniform conductivity tensor ${\overleftrightarrow{\sigma }}^{(m)}(z^{\prime} ,z^{\prime\prime} )$ entering Eq. 5 is extracted from the current-density operator calculated in the framework of a microscopic density matrix approach and is composed of the nonlocal paramagnetic conductivity tensor and the local isotropic diamagnetic conductivity tensor. The conductivity tensor can be either determined by numerically solving the Heisenberg Equation in which case higher order contributions (microscopic scattering, many-body interactions, etc.) can be included on equal footing or in its simplest form derived analytically (as done here):

6 $${\sigma }_{ij}(z,z^{\prime} )=-\,\frac{2{\rm{i}}{e}^{2}\hslash }{\omega }\sum _{\lambda ,\lambda ^{\prime} }\sum _{{\bf{k}}}\frac{({n}_{{\bf{k}}}^{\lambda ^{\prime} }-{n}_{{\bf{k}}}^{\lambda })}{(\omega +{\rm{i}}{\gamma }_{\lambda \lambda ^{\prime} }+{\omega }_{{\bf{k}}}^{\lambda \lambda ^{\prime} })}{{\rm{f}}}_{\lambda \lambda ^{\prime} }(z^{\prime} ){{\rm{f}}}_{\lambda \lambda ^{\prime} }(z){k}_{i}{k}_{j}$$

7 $$+\,\frac{2{\rm{i}}{e}^{2}}{\omega }\sum _{\lambda }\sum _{{\bf{k}}}{n}_{{\bf{k}}}^{\lambda }{{\rm{f}}}_{\lambda \lambda }(z^{\prime} )\delta (z-z^{\prime} ){\delta }_{ij}\,\,\forall i,j=x,y$$

8 $${\sigma }_{iz}(z,z^{\prime} )=-\,{\sigma }_{zi}^{\mathrm{(1)}}(z^{\prime} ,z)$$

9 $$=\,\frac{{e}^{2}\hslash }{\omega }\sum _{\lambda ,\lambda ^{\prime} }\sum _{{\bf{k}}}\frac{({n}_{{\bf{k}}}^{\lambda ^{\prime} }-{n}_{{\bf{k}}}^{\lambda })}{(\omega +{\rm{i}}{\gamma }_{\lambda \lambda ^{\prime} }+{\omega }_{{\bf{k}}}^{\lambda \lambda ^{\prime} })}{{\rm{g}}}_{\lambda \lambda ^{\prime} }(z^{\prime} ){{\rm{f}}}_{\lambda \lambda ^{\prime} }(z){k}_{i}\,\,\forall \,i=x,y$$

10 $${\sigma }_{zz}(z,z^{\prime} )=-\,{\rm{i}}{e}^{2}\hslash \sum _{\lambda ^{\prime}  > \lambda ^{\prime} }\frac{\omega }{{({\omega }_{0}^{\lambda \lambda ^{\prime} })}^{2}}\sum _{{\bf{k}}}\frac{{\omega }_{{\bf{k}}}^{\lambda \lambda ^{\prime} }({n}_{{\bf{k}}}^{\lambda ^{\prime} }-{n}_{{\bf{k}}}^{\lambda })}{{(\omega +{\rm{i}}{\gamma }_{\lambda \lambda ^{\prime} })}^{2}-{({\omega }_{{\bf{k}}}^{\lambda \lambda ^{\prime} })}^{2}}{{\rm{g}}}_{\lambda \lambda ^{\prime} }(z^{\prime} ){{\rm{g}}}_{\lambda \lambda ^{\prime} }(z)$$

with

11 $${{\rm{f}}}_{\lambda \lambda ^{\prime} }(z)=\frac{1}{2}(\frac{1}{m({\varepsilon }_{0}^{\lambda },z)}+\frac{1}{m({\varepsilon }_{0}^{\lambda ^{\prime} },z)}){\varphi }_{\lambda }(z){\varphi }_{\lambda ^{\prime} }(z)$$

12 $${{\rm{g}}}_{\lambda \lambda ^{\prime} }(z)={\varphi }_{\lambda }(z)\frac{1}{m({\varepsilon }_{0}^{\lambda ^{\prime} },z)}\frac{d}{dz}{\varphi }_{\lambda ^{\prime} }(z)-{\varphi }_{\lambda ^{\prime} }(z)\frac{1}{m({\varepsilon }_{0}^{\lambda },z)}\frac{d}{dz}{\varphi }_{\lambda }(z\mathrm{).}$$

Here, ${n}_{{\bf{k}}}^{\lambda }$ denotes electron distribution at in-plane momentum ${\bf{k}}$, ${\omega }_{{\bf{k}}}^{\lambda \lambda ^{\prime} }$ the transition frequency between subbands $\lambda$ and $\lambda ^{\prime}$, ${\varphi }_{\lambda }(z)={\varphi }_{\lambda ,c}(z)$ is the conduction band component of the envelope wave function in the confined direction, and $m({\varepsilon }_{0}^{\lambda },z)$ the energy- and space-dependent effective mass of subband $\lambda$. Once the local electric field is determined we obtain the absorptivity

13 $$A=1-|{E}_{R}/{E}_{0}{|}^{2}-|{E}_{T}/{E}_{0}{|}^{2},$$

where ${E}_{R}$ and ${E}_{T}$ are the reflected and transmitted fields in the full multilayer geometry. Calculating the local displacement field using

14 $${{\bf{D}}}^{(n)}(z)={\varepsilon }_{0}{\varepsilon }_{r}^{(n)}{{\bf{E}}}^{(n)}(z)+\frac{{\rm{i}}}{\omega }\int dz^{\prime} {\overleftrightarrow{\sigma }}^{(n)}(z,z^{\prime} ,\omega )\cdot {{\bf{E}}}^{(n)}(z^{\prime} ,\omega ),$$

we can now extract the permittivities using

15 $${\varepsilon }_{ii}^{(S)}=\frac{{\int }_{S}dz{D}_{i}(z)}{{\varepsilon }_{0}{\int }_{S}dz{E}_{i}(z)}\,i=x,y,z\mathrm{.}$$

In Eq. 15 $S$ denotes the specific region that is being averaged over. Averaging over the quantum well and barrier layers separately ($S=1,\mathrm{..},N)$ allows the extraction of the anisotropic permittivities for the individual layers that can be used for electromagnetic modeling of SHMs in the framework of a transfer-matrix method (anisotropic superlattice model). Averaging instead over the full SHM ($S=SHM$) allows the extraction of effective anisotropic permittivities that allow to describe the homogenized SHM (anisotropic effective medium model).

## Results

In order to test the proposed theory we performed a study investigating the impact of the thickness of the barrier separating a number of doped quantum wells in SHMs. We grew four different samples composed by alternating ${N}_{QW}$ pairs of 20-nm-thick highly doped In_0.53_Ga_0.47_As and L_*B*_-nm-thick undoped In_0.52_Al_0.48_As layers. Hall measurements showed the average electron density on the order of $1.9\times {10}^{19}\,{{\rm{cm}}}^{-3}$, ellipsometry data and simulations indicated an optical active carrier density of $\approx 1.3\times {10}^{19}\,{{\rm{cm}}}^{-3}$. The number of quantum wells N_*QW*_ is chosen to keep the sample thickness constant. The samples were grown using molecular beam epitaxy on a 0.65-mm-thick InP substrate with a 200 nm thick In_0.52_Al_0.48_As buffer layer. Growth characteristics of the four samples are given in Table 1.

**Table 1:** Characteristics of the four samples used to study the impact of barrier thickness on the electromagnetic response of SHMs.

Sample	L_*W*_ (*nm*)	L_*B*_ (*nm*)	N_*QW*_
EB4910	20	40	36
EB4908	20	70	24
EB4907	20	100	18
EB4906	20	200	10

**Figure 1:**
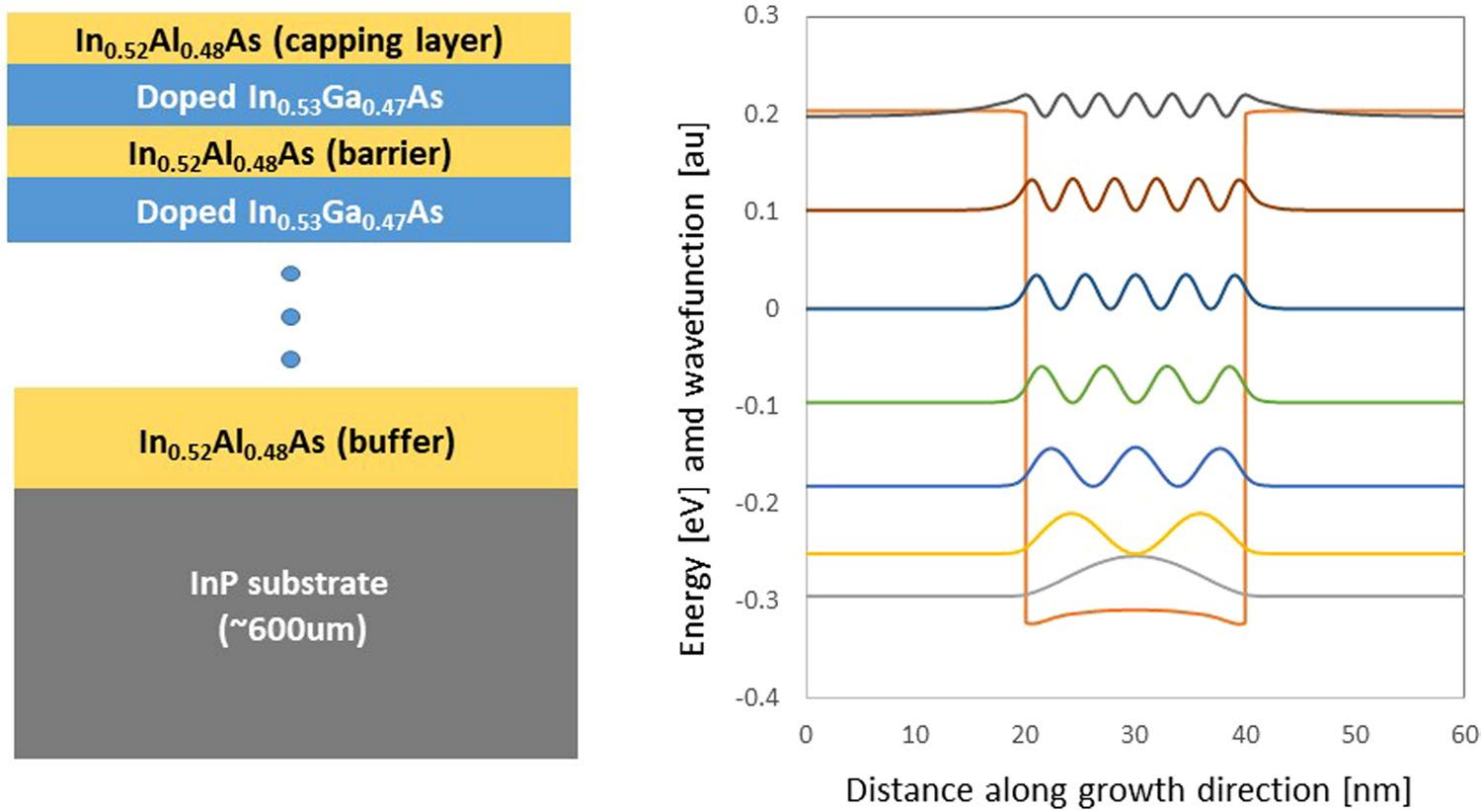
Schematic of the fabricated structure and calculated band structure for one period of sample EB4910. The Fermi level is taken to be 0 eV.

**Figure 2:**
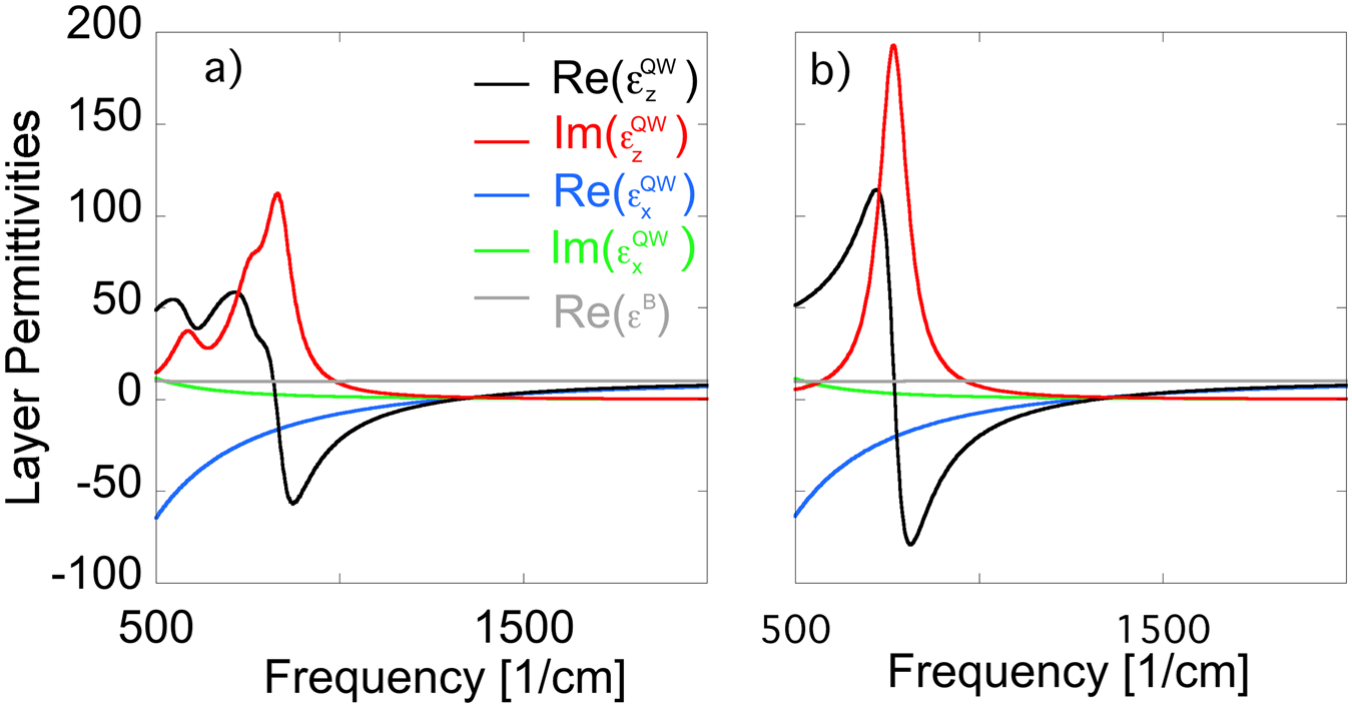
(**a**) Calculated permittivity functions for the layers composing SHM EB4910 using our new microscopic theory. (**b**) Permittivity functions for the individual quantum well and barrier layers composing SHM EB4910 extracted via ellipsometry. The presented functions were extracted by fitting the ellipsometry data assuming a Lorentz function for the out-of-plane permittivity of the quantum well layer and Drude functions for the in-plane permittivities of the quantum well layer.

We first calculated the band structure of each sample using a fully-coupled 8  ×  8 ${\bf{k}}\cdot {\bf{p}}$ Hamiltonian^41^ that includes the light-hole, heavy-hole, split-off-hole and the lowest s-like conduction bands. To obtain correct band dispersions the impact of remote bands has been included via the Löwdin renormalization^42^. The screening potential due to carrier charges is incorporated by iteratively solving the Schrödinger Equation (using the ultimate concept approach^43,44^) and Poisson’s equation (using a predictor-corrector approach^45^). The calculated band structure for one period of sample EB4910 is shown in Fig. 1. Note, that the band structures are identical for all samples since only the barrier thickness and number of quantum wells differ between the samples. In Fig. 2(a) we show the calculated permittivity functions for the individual layers composing SHM EB4910. The epsilon-near-zero (ENZ) point of the in-plane permittivity (${\varepsilon }_{x}^{QW}$) occurs at $\approx 1290$ cm^−1^ while that of the out-of-plane permittivity (${\varepsilon }_{z}^{QW}$) occurs at $\approx 1325$ cm^−1^. For low frequencies the permittivity of the QW well layer is very anisotropic. The in-plane permittivity is Drude-like whereas the out-of-plane permittivity is Lorentz-like.

In Fig. 2(b) we present the corresponding permittivity functions that were extracted via ellipsometry (anisotropic superlattice model). In previous work^34^ we obtained the anisotropic permittivity functions of the individual layers by fitting the ellipsometry data to anisotropic Drude functions. Guided by the theoretical results, we here extracted the permittivity functions by fitting the ellipsometry data assuming a Lorentz function for the out-of-plane permittivity of the quantum well layer and Drude functions for the in-plane permittivities. Furthermore, we imposed the constraint that in-plane and out-of-plane permittivities of the quantum well layer converge at high frequencies. As can be seen by comparing Fig. 2(a,b), the ENZ points of both in-plane and out-of-plane permittivities are almost identical compared to the calculated permittivities. However, the out-of-plane permittivities have different shapes for the very low frequency range. This is due to the fact that the calculated permittivity reflects the existence of multiple ISB transitions. The experimentally extracted function, however, was obtained through a fit to a single transition Lorentz function. For this reason, the observed differences in the low frequency range are not surprising.

**Figure 3:**
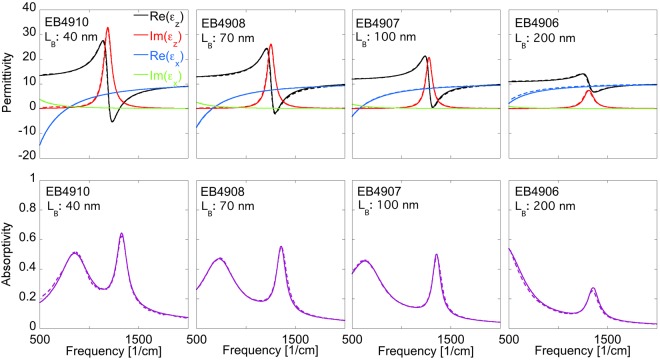
Effective permittivities describing the homogenized SHM (top row) and the corresponding p-polarized absorptivity spectra (bottom row) for all 4 samples at an angle of incidence of 55°. Results using the anisotropic effective medium approach with permittivities extracted from spectroscopic ellipsometry are presented with solid lines whereas the results from the microscopic theory are shown with dashed lines.

In Fig. 3 we next show the corresponding effective permittivities describing the homogenized SHM (top row) and the corresponding p-polarized absorptivity spectra (bottom row) for all 4 samples at an angle of incidence of 55 deg. Results using the anisotropic effective medium approach with permittivities extracted from spectroscopic ellipsometry are presented with solid lines whereas the results from the microscopic theory are shown with dashed lines. Interestingly enough, although the experimental and theoretical out-of-plane permittivities of the quantum well layer differ in the low frequency range, the effective permittivities describing the full SHM are almost undistinguishable. The low frequency absorptivity peak is associated with ENZ of the effective in-plane permittivity. With increasing barrier thickness the ENZ frequency for ${\varepsilon }_{\parallel }$ is shifted to lower frequencies, whereas the occurrence of the ENZ mode for ${\varepsilon }_{\perp }$ gradually disappears. These results highlight the great design freedom for SHMs. Already by changing the barrier thickness we can tune the dielectric response of the SHM even though the electromagnetic response of the individual layers is the same.

**Figure 4:**
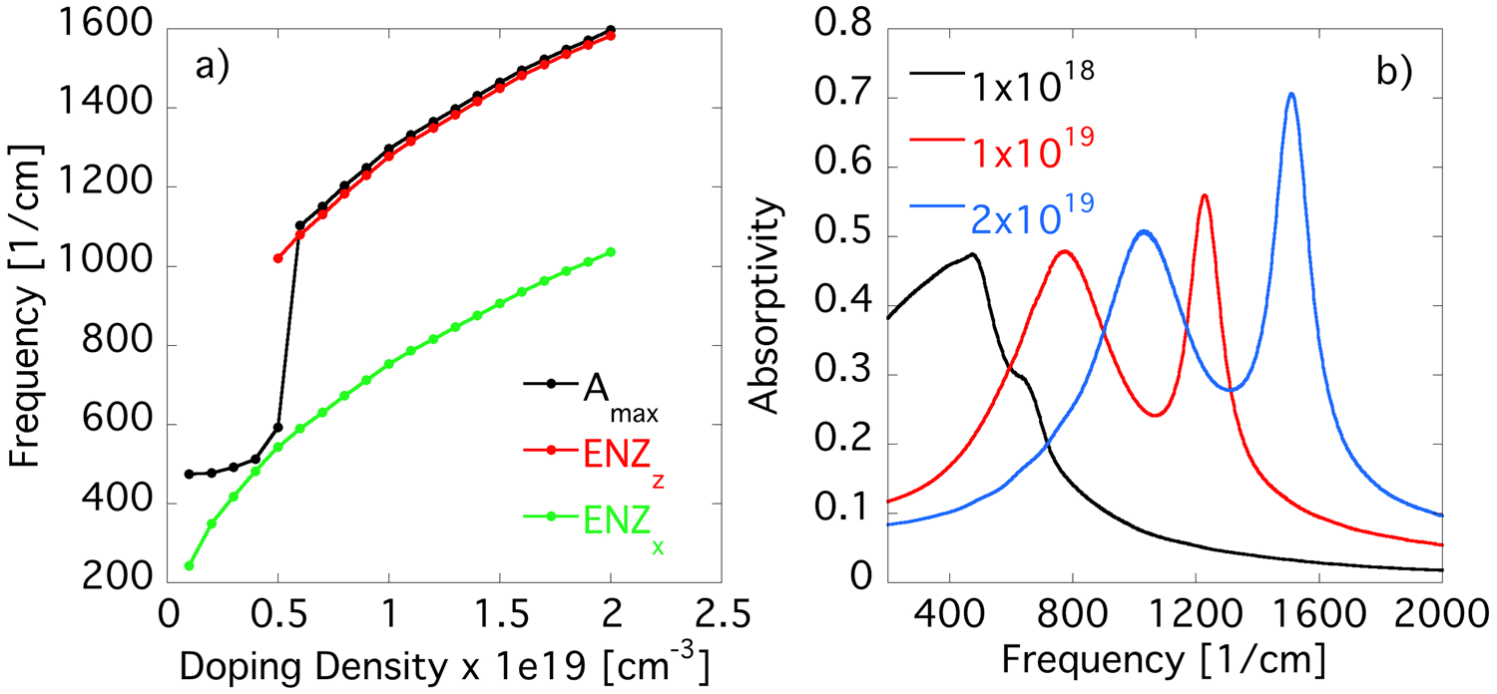
(**a**) Dependence of absorptivity peak of the SHM and ENZ points of effective in-plane and out-of-plane permittivities on doping density. (**b**) Absorptivity spectrum of SHM for three selected doping densities. As soon as the superlattice supports ENZ of ${\varepsilon }_{z}^{eff}$, the absorptivity peak of the SHM is directly connected to the effective out-of-plane permittivity.

As stated previously, the electromagnetic response of a quantum well can be altered dramatically through bandstructure engineering and can depend strongly on confinement effects, doping, and many-body interactions, which of course will alter the electromagnetic response of the SHM. To demonstrate the dependence of the electromagnetic behavior on doping we present in Fig. 4(a) the calculated dependence of the ENZ points of the effective permittivies and peak absorptivity of the SHM on carrier density and in Fig. 4(b) the corresponding absorptivity spectrum for three selected doping densities. To obtain the data plotted in Fig. 4 we simulated a series of samples where we tuned the doping density in SHM EB4910 from $0.1\times {10}^{19}$−$2\times {10}^{19}$ cm^3^. As can be seen, as soon as the superlattice supports an ENZ condition of the effective out-of-plane permittivity, the ENZ point of ${\varepsilon }_{z}^{eff}$ is directly connected to the absorption peak of the SHM.

Last, we investigate the applicability of the effective conductivity model for the SHMs considered in this study. Since InAlAs/InGaAs quantum wells exhibit considerable nonparabolicty effects we modified the ECM model by introducing the k-dependent plasma frequency

16 $${\omega }_{p,n}^{2}(k)=\frac{{f}_{n}{e}^{2}{\rm{\Delta }}{N}_{n}(k)}{{m}^{\ast }{\varepsilon }_{0}{\varepsilon }_{r}{L}_{eff,n}(k)}$$

and k-dependent effective length

17 $${L}_{eff,n}(k)=\frac{{\omega }_{n}^{2}}{{\omega }_{n}^{2}(k)}{L}_{eff,n}\mathrm{.}$$

**Figure 5:**
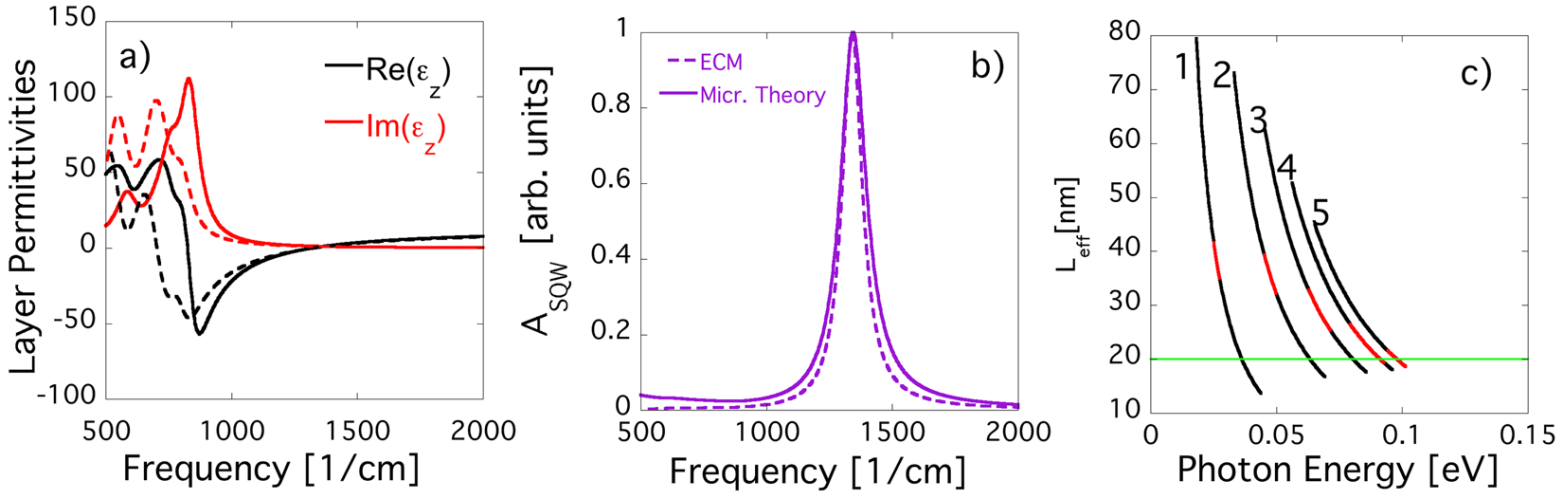
(**a**) Permittivity functions for the quantum well layer calculated using the effective conductivity model (${\varepsilon }_{\perp }^{ECM}$, dashed lines) and the full theory (${\varepsilon }_{\perp }^{QW}$, solid lines). (**b**) Optical absorption of a single quantum well per unit area calculated using either our new microscopic theory (solid line) or the effective conductivity model (calculated using Eq. 2, dashed line). (**c**) Dependence of ${L}_{eff,n}$ on photon energy. Only the energy ranges indicated in red are optically active. Outside of these energy ranges, optical transitions are forbidden due to Pauli blocking effects.

In this modified ECM model, both the plasma frequency as well as the effective thickness now carry an additional dependence on in-plane momentum (or photon energy), which was not accounted for in Eq. 4. In Fig. 5(a) we compare the out-of-plane permittivity of the quantum well layer of sample EB4910 to the effective permittivity obtained in the effective conductivity model (modified to account for nonparabolicity effects as described above). As can be seen, the ENZ point of the out-of-plane permittivity is almost identical and both models show strong deviations from a Drude function for the low frequency regime. The observed differences between the two models are due to the omission of retardation effects, restriction to transitions between consecutive levels only, and other approximations that were employed in the derivation of the effective conductivity model. Despite the differences in the low frequency regime the optical absorption of a single quantum well per unit area differs only very slightly between the models which again shows that absorption spectra alone are not sensitive enough to fully capture the detailed electromagnetic response of SHMs such as the impact of retardation effects. Note, that the absorption shown here is only the absorption of a single quantum well per unit area and not the absorption per period as the barrier thickness is not accounted for in Eq. 2. The observed agreement between the calculated permittivities might suggest that the ECM model can be used reliably to determine effective permittivities of SHM. However, whereas the permittivity shown in Fig. 5 (a, solid lines) is the permittivity of a well layer with thickness 20 nm, the permittivity shown in Fig. 5 (a, dashed lines) is actually only the approximated permittivity of a layer of quantum well material with thickness **L**_**eff**_. This effective thickness can differ from the physical thickness of the quantum well and as mentioned above now depends not only on the optical transition but also the photon energy. To illustrate this dependence we show in Fig. 5(c) the dependence of ${L}_{eff,n}$ on photon energy and transition number. The physical thickness of the quantum well is 20 nm (green line), however, ${L}_{eff}$ varies quite strongly between the different ISB transitions. Since the sample is highly doped, Pauli blocking effects yield only a small energy range for each transition where the plasma frequency is unequal 0 (shown in red). Outside of these energy ranges, optical transitions are forbidden due to Pauli blocking effects. As can be seen in Fig. 5(c), for the optically active energy range of transitions 4 and 5, the corresponding effective thicknesses ${L}_{eff\mathrm{,4}}$ and ${L}_{eff\mathrm{,5}}$ overlap with the actual physical thickness of the well. Other transitions, however, have quite different effective thicknesses. This dependence of ${L}_{eff}$ on transition number and photon energy introduces an ambiguity on how to best extract effective permittivities for the SHM. In the case presented here, the results from our microscopic theory show that ${L}_{eff}={L}_{QW}$ is a good approximation. However, it has to be kept in mind that this approximation might only be valid for the SHM considered in this work. Furthermore, it has to be stressed that the ECM will ultimately fail to correctly capture the quantum well permittivity if the SHM contains more complicated quantum well structures where the permittivity is not dominated by transitions between consecutive energy levels.

## Conclusion

We have studied semiconductor hyperbolic metamaterials (SHMs) at the quantum limit experimentally using spectroscopic ellipsometry as well as theoretically using a new microscopic theory. Comparison between theory and experiments has shown that our theory correctly captures light-matter interactions in SHMs at the quantum limit. The presented theory predicts absorptivity of the full multilayer system and for the first time allows the prediction of in-plane and out-of-plane dielectric functions for every individual layer constructing the SHM as well as effective dielectric functions that can be used to describe the homogenized SHM. Our results show that the absorptivity peak of the SHM can be extrapolated from the effective out-of-plane permittivity of the SHM as soon as the ENZ condition of ${\varepsilon }_{z}^{eff}$ is fulfilled. The theory allows to either determine the microscopic conductivity tensor describing the material response approximately in a simple analytical form or numerically in which case higher order contributions (microscopic scattering, many-body interactions, etc.) can be included on equal footing. Studies investigating the impact of many-body interactions on SHMs are under way and will be published in a future paper. Besides validating the presented theory, we also investigated the applicability of the widely-used effective conductivity model to the description of SHMs and found that the model captures the important physics well in the SHMs considered in this work. However, caution should be used when extending the ECM to more complicated SHMs. For more complicated SHMs, where the ECM will ultimately fail, the presented microscopic theory has to be used to ensure that the underlying physics are captured correctly.

## Data Availability

The datasets generated during the current study are available from the corresponding author on reasonable request.
